# Factors associated with low adherence to medicine treatment for chronic diseases in Brazil

**DOI:** 10.1590/S1518-8787.2016050006150

**Published:** 2016-12-01

**Authors:** Noemia Urruth Leão Tavares, Andréa Dâmaso Bertoldi, Sotero Serrate Mengue, Paulo Sergio Dourado Arrais, Vera Lucia Luiza, Maria Auxiliadora Oliveira, Luiz Roberto Ramos, Mareni Rocha Farias, Tatiane da Silva Dal Pizzol

**Affiliations:** I Departamento de Farmácia. Faculdade de Ciências da Saúde. Universidade de Brasília. Brasília, DF, Brasil; II Departamento de Medicina Social. Faculdade de Medicina. Universidade Federal de Pelotas. Pelotas, RS, Brasil; III Programa de Pós-Graduação em Epidemiologia. Universidade Federal do Rio Grande do Sul. Porto Alegre, RS, Brasil; IV Departamento de Farmácia. Faculdade de Farmácia, Odontologia e Enfermagem. Universidade Federal do Ceará. Fortaleza, CE, Brasil; V Departamento de Política de Medicamentos e Assistência Farmacêutica. Escola Nacional de Saúde Pública Sérgio Arouca. Fundação Oswaldo Cruz. Rio de Janeiro, RJ, Brasil; VI Departamento de Medicina Preventiva. Escola Paulista de Medicina. Universidade Federal de São Paulo. São Paulo, SP, Brasil; VII Departamento de Ciências Farmacêuticas, Centro de Ciências da Saúde. Universidade Federal de Santa Catarina. Florianópolis, SC, Brasil; VIII Departamento de Produção e Controle de Medicamentos. Faculdade de Farmácia. Universidade Federal do Rio Grande do Sul. Porto Alegre, RS, Brasil

**Keywords:** Patient Dropouts, Medication Adherence, Drugs of Continuous Use, Chronic Disease, Health Services Accessibility, Socioeconomic Factors, Health Surveys

## Abstract

**OBJECTIVE:**

To analyze factors associated with low adherence to drug treatment for chronic diseases in Brazil.

**METHODS:**

Analysis of data from *Pesquisa Nacional sobre Acesso, Utilização e Promoção do Uso Racional de Medicamentos* (PNAUM - Brazilian Survey on Access, Use and Promotion of Rational Use of Medicines), a population-based cross-sectional household survey, based on a probabilistic sample of the Brazilian population. We analyzed the association between low adherence to drug treatment measured by the Brief Medication Questionnaire and demographic, socioeconomic, health, care and prescription factors. We used Poisson regression model to estimate crude and adjusted prevalence ratios, their respective 95% confidence interval (95%CI) and p-value (Wald test).

**RESULTS:**

The prevalence of low adherence to drug treatment for chronic diseases was 30.8% (95%CI 28.8-33.0). The highest prevalence of low adherence was associated with individuals: young adults; no education; resident in the Northeast and Midwest Regions of Brazil; paying part of the treatment; poor self-perceived health; three or more diseases; reported limitations caused by a chronic disease; using five drugs or more.

**CONCLUSIONS:**

Low adherence to drug treatment for chronic diseases in Brazil is relevant, and regional and demographic differences and those related to patients’ health care and therapy regime require coordinated action between health professionals, researchers, managers and policy makers.

## RESUMO

**OBJETIVO:**

Analisar fatores associados à baixa adesão ao tratamento farmacológico de doenças crônicas no Brasil.

**MÉTODOS:**

Análise de dados oriundos da Pesquisa Nacional sobre Acesso, Utilização e Promoção do Uso Racional de Medicamentos (PNAUM), inquérito domiciliar de base populacional, de delineamento transversal, baseado em amostra probabilística da população brasileira. Analisou-se a associação entre baixa adesão ao tratamento medicamentoso mensurado pelo *Brief Medication Questionnaire* e fatores demográficos, socioeconômicos, de saúde, assistência e prescrição. Foi utilizado modelo de regressão de Poisson para estimar as razões de prevalência brutas e ajustadas, os respectivos intervalos de 95% de confiança (IC95%) e p-valor (teste de Wald).

**RESULTADOS:**

A prevalência de baixa adesão ao tratamento farmacológico de doenças crônicas foi de 30,8% (IC95% 28,8–33,0). As maiores prevalências de baixa adesão estiveram associadas a indivíduos: adultos jovens; que nunca estudaram; residentes na região Nordeste e Centro-Oeste do País; que tiveram que pagar parte do tratamento; com pior autopercepção da saúde; com três ou mais doenças; que referiam limitação causada por uma das doenças crônicas; e que faziam uso de cinco medicamentos ou mais.

**CONCLUSÕES:**

A baixa adesão ao tratamento medicamentoso para doenças crônicas no Brasil é relevante e as diferenças regionais, demográficas e aquelas relacionadas à atenção à saúde do paciente e ao regime terapêutico requerem ações coordenadas entre profissionais de saúde, pesquisadores, gestores e formuladores de políticas para o seu enfrentamento.

## INTRODUCTION

Noncommunicable diseases, a global health problem, are the target of various prevention and control programs and initiatives^1^. Many noncommunicable diseases can be controlled by the use of drugs, which, when available and properly used, lead to therapeutic success. An important factor that directly influences therapeutic outcomes is adherence to drug treatment, defined as the degree of agreement between a person’s behavior and professional guidance^22^.

Factors related to non-adherence to treatment described in the literature concern individual characteristics of patients, the actual disease, the drugs used and interaction between patients and health services, among others^20^. The characteristics of certain health conditions or therapies may lead to specific barriers to adherence. For some asymptomatic diseases such as high blood pressure, patients may have difficulty to use drugs regularly because of the lack of visible symptoms or understanding of the disease’s behavior^15^. For illnesses that require complex regimes (polypharmacy, multiple daily administrations, difficulties with administration), such as asthma and diabetes, the actual daily difficulties associated with the use of drugs are an important barrier to adherence^3^.

According to the World Health Organization (WHO), non-adherence to long-term treatment in the population at large is around 50.0%^20^. In a systematic review summarizing studies published over 50 years, DiMatteo^5^ (2004) identified an average non-adherence rate of 24.8%.

Brazil lacks sufficient evidence on the prevalence of low adherence among patients with chronic diseases based on nationwide studies. The available studies used local or regional samples^17^, population subgroups (such as older adults)^20^, or focused on specific chronic diseases such as high blood pressure^6^ ^,^ ^7^ ^,^ ^18^. Therefore, studies that estimate treatment adherence among the Brazilian population with chronic diseases are important to support health policy and practice aimed at improving access to and rational use of drugs.

The aim of this study was to analyze factors associated with low adherence to drug treatment for chronic diseases in Brazil.

## METHODS

The data analyzed in this study are from *Pesquisa Nacional sobre Acesso, Utilização e Promoção do Uso Racional de Medicamentos* (PNAUM – National Survey on Access, Use and Promotion of Rational Use of Medicines), a population-based cross-sectional household survey, based on a probabilistic sample of the Brazilian population. Data were collected from September 2013 to February 2014. The study population lived in permanent private households in urban areas of Brazil, and included individuals of all ages. Face-to-face interviews were carried out in households using questionnaires, and data were collected and stored in electronic devices. The tools were developed by a group of expert researchers from Brazilian universities and standardized and tested before being administered.

The complex sampling process resulted in a sample that ensured national representation for the five Brazilian regions, stratified by gender and age groups. Further details on sampling and data collection can be found in the PNAUM methodology article^13^. This study included adults aged 20 or older who reported at least one chronic disease diagnosed at least six months prior to the interview (n = 14,358). The investigation on adherence to drug treatment included all subjects who reported medical indication for treatment and were using medication for the chronic diseases mentioned at the interview (n = 11,842).

To assess adherence reported by patients, we used the Brief Medication Questionnaire (BMQ), composed of three areas that identify barriers to adherence related to the regime, beliefs, and memories of the drug treatment. We used the BMQ^2^ version translated into Portuguese, which classifies individuals into four categories of adherence to treatment, according to the number of positive responses in any of the areas: high adherence (none), likely high adherence (1), likely low adherence (2), and low adherence (3 or more). The outcome analyzed in this study was prevalence of low adherence to treatment, considered as a score of 2 or more in any field.

The variables related to demographic and socioeconomic characteristics were: gender (female; male); age group (20-39; 40-59; 60 and over); self-reported skin color (white, non-white), marital status (with partner; without partner); education reported in grades and reclassified in years of study (0, 1-8, 8 years or more); economic status according to *Critério de Classificação Econômica Brazil* (Brazilian Economic Classification Criterion) of *Associação Brasileira de Empresas de Pesquisa* (ABEP – Brazilian Association of Survey Companies [A/B; C; D/E]), geographic region of residence (North, Northeast, Southeast, South, Midwest) and whether the respondent had health insurance.

Regarding health care, we analyzed the number of hospitalizations and emergency visits in the previous year (none, one, two or more), if the individual visits and has a single doctor to treat the diseases, and free access to medicines (all drugs; any drug; no drugs).

Regarding perception of health and morbidities, the following variables were evaluated: number of chronic diseases reported (high blood pressure; diabetes; stroke; lung disease; depression; rheumatism; other chronic diseases lasting more than six months) grouped into one, two, three or more conditions; self-perceived health, analyzed in five categories (very poor; poor; average; good; very good); and reported limitations related to at least one chronic disease. Regarding drug use, we analyzed the number of drugs used (continuous or occasional) (1; 2; 3 or 4; 5 or more).

The analyses were performed with Stata version 11.0 software, using the appropriate set of svy commands to analyze complex samples and ensuring the necessary weighting, considering the sample design. Exploratory descriptive analysis was performed for all the variables involved in the study, presenting the relative frequencies and respective 95% confidence intervals (95%CI). For the univariate analysis, the BMQ score was dichotomized, considering as low adherence a score of two or more. In the crude analysis, the prevalence of low adherence to treatment was calculated for the categories of the independent variables, considering the dichotomous outcome. A 5% significance level was adopted.

We used Poisson regression model to estimate crude and adjusted prevalence ratios (PR) and 95%CI, considering the effect of the sample design with Stata svy commands. We attempted to control possible confounding factors in the multivariate analysis, using a hierarchical analysis model (Figure). Variables with p < 0.20 were included in the multivariate model, and we adopted a significance level of 5% to retain variables in the model, with “backward” selection of variables. The statistical significance of prevalence ratios obtained in Poisson regression models was assessed by the Wald test.

**Figure 1:**
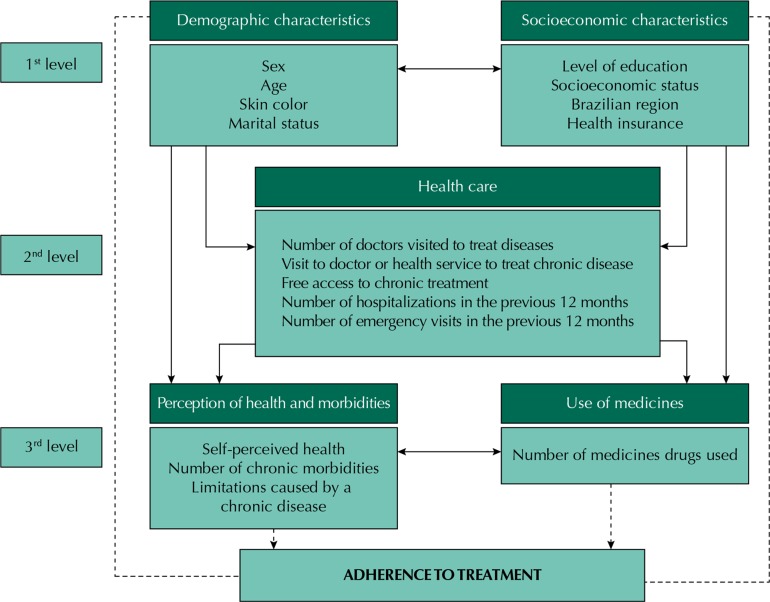
Hierarchical model for the analysis of factors associated to adherence to drug therapy for chronic diseases in Brazil. PNAUM, Brazil, 2014.

The study was approved by the *Comissão Nacional de* Ética *em Pesquisa* (National Research Ethics Committee – Opinion 398,131, of September 6, 2013). All interviews were conducted after the respondents or their legal representatives had read and signed the consent form.

## RESULTS

Table 1 shows the adherence classification according to BMQ. The prevalence of low adherence (dichotomized score) to drug treatment for chronic diseases in Brazil was 30.8% (95%CI 28.8-33.0) and only 2.6% (95%CI 2.1-3.2) of respondents were classified as adhering to prescribed therapies (no positive response in the evaluated areas).

**Table 1:** Classification of adherence to treatment for chronic diseases by adults aged 20 or over in Brazila. PNAUM, Brazil, 2014. (N = 11,842)

BMQ^a^	%^b^	95%CI
Categorical score		
Adherence	2.6	2.1–3.2
Likely adherence	66.6	64.4–68.7
Likely low adherence	17.0	15.5–18.6
Low adherence	13.8	12.7–15.1
Dichotomous BMQ score		
Adherence or likely adherence	69.2	67.0–71.2
Likely low adherence or low adherence	30.8	28.8–33.0

^a^ According to Brief Medication Questionnaire (BMQ).

^b^ Percentage adjusted by sample weighting and post-stratification by age and gender.

The sample breakdown and prevalence of low adherence in relation to socioeconomic and demographic characteristics are shown in Table 2. Prevalence of low adherence (statistically significant) was higher in the following categories: younger individuals (20-39 years) and those residing in the Northeast and Midwest Regions. Prevalence of low adherence was also higher, although not statistically significant, among individuals who were female, non-white, reported not having a partner and having no education, D/E economic status, and without health insurance.

**Table 2:** Prevalence of low adherencea,b to treatment for chronic diseases by adults aged 20 or over in Brazil, by demographic and socioeconomic characteristics. PNAUM, Brazil, 2014. (N = 11,842)

Variable	Breakdown in sample		Prevalence of low adherence^c^	
	
	%^a^	95%IC	%^a^	95%IC
Demographic characteristics				
Gender				
Male	35.3	33.9–36.7	28.3	25.4–31.5
Female	64.7	63.3–66.1	32.1	29.9–34.4
Age (years)				
20-39	15.0	13.6–16.6	38.6	33.1–44.5
40-59	42.8	41.1–44.4	30.3	27.8–33.0
≥ 60	42.2	40.4–44.0	28.7	26.5–31.0
Skin color				
White	50.6	47.8–53.4	30.0	27.3–32.8
Non-white	49.4	46.6–52.2	31.4	28.9–34.0
Marital status				
With partner	61.4	59.8–62.9	30.5	28.3–32.8
Without partner	38.6	37.1–40.2	31.6	28.9–34.5
Socioeconomic characteristics				
Level of education (years of study)				
No education	15.0	13.7–16.4	35.5	31.5–39.8
1 to 8 years	43.1	41.2–45.0	28.5	25.8–31.3
≥ 8 years	41.8	40.0–43.7	31.7	29.3–34.2
Economic status ABEP^d^				
A/B	24.6	22.3–27.0	30.6	26.9–34.6
C	54.8	52.9–56.7	30.3	28.0–32.7
D/E	20.7	18.9–22.5	32.6	29.2–36.2
Brazilian region				
North	4.2	3.2–5.3	22.7	18.8–27.1
Northeast	20.6	16.8–25.0	38.1	35.1–41.2
Southeast	51.9	46.0–57.8	29.2	25.7–33.0
South	15.7	12.7–19.3	26.8	24.5–29.2
Midwest	7.7	6.0–9.8	35.5	31.6–39.6
Health plan				
Yes	28.4	26.0–31.0	29.0	26.1–32.1
No	71.6	69.0–74.0	31.6	29.3–34.0

Total			30.8	28.8–33.0

^a^ According to *Brief Medication Questionnaire* (BMQ).

^b^ Percentage adjusted by sample weighting and post-stratification by age and gender.

^c^ Non-adherence = low adherence according to BMQ (2 or more positive answers).

^d^ According to *Critério de Classificação Econômica Brasil* 2013 – ABEP (www.abep.org).

The characteristics of individuals with low adherence to treatment related to health care, self-perception and morbidities, and the use of drugs, are presented in Table 3. Prevalence of low adherence was higher in those individuals who do not see a doctor to treat chronic diseases, although it was not statistically significant. On the other hand, individuals visiting more than one doctor to treat those diseases had a 47.0% higher likelihood of low adherence to treatment than those who had only one doctor. Those who had to pay part of the treatment, had two or more hospitalizations, or received emergency care in the previous year had an 80.0% lower adherence to treatment, approximately.

**Table 3:** Prevalence of low adherencea,b to treatment for chronic diseases by adults aged 20 or over in Brazil, by characteristics related to health care, health perception, morbidities and drug use. PNAUM, Brazil, 2014. (N = 11,842)

Variable	Sample breakdown		Prevalence of low adherence	
	
	%	95%IC	%	95%IC
Health system characteristics				
Visits doctor to treat chronic diseases				
Yes	93.3	92.3–94.2	31.2	29.0–33.5
No	6.7	5.8–7.7	32.3	27.9–37.0
Number of doctors visited to treat chronic diseases				
One	68.2	66.3–70.0	27.2	24.9–29.6
More than one	31.8	30.0–33.7	40.1	37.2–43.2
Free access to chronic drug therapy for chronic diseases				
All free	46.9	44.5–49.2	25.4	23.0–28.1
Any free	20.3	19.1–21.5	46.6	42.7–50.7
None free	32.9	30.8–35.1	29.0	26.5–31.7
Number of hospitalizations in the previous 12 months				
None	89.1	88.2–90.0	29.8	27.7–32.1
1	8.2	7.5–9.0	34.6	29.8–39.8
2 or more	2.7	2.2–3.2	51.8	42.7–60.9
Number of emergency visits in the previous 12 months				
None	77.0	75.3–78.6	27.1	25.0–29.3
1	14.9	13.8–16.0	39.8	36.2–43.7
2 or more	8.1	7.2–9.1	50.0	44.6–55.4
Self-perceived health and morbidities				
Self-perceived health				
Very good	5.1	4.4–5.9	17.2	11.4–25.1
Good	45.6	43.6–47.6	25.0	22.6–27.7
Average	40.8	39.1–42.4	35.9	33.4–38.5
Poor	6.3	5.6–7.0	46.4	41.1–51.7
Very poor	2.3	1.9–2.7	49.6	39.7–59.5
Number of chronic diseases (comorbidities)				
1	44.8	42.9–46.7	20.9	18.8–23.1
2	27.2	26.1–28.4	33.3	30.6–36.1
3 or more	28.0	26.3–29.7	44.2	41.0–47.4
Limitations due to chronic disease				
No limitations	48.5	46.7–50.3	21.9	19.9–24.1
Limitations	51.5	49.7–53.3	39.5	36.9–42.2
Use of drugs				
Number of drugs used (continuous or occasional)				
1	34.6	33.2–36.0	20.6	18.2–23.2
2	26.0	24.7–27.3	26.4	23.8–29.2
3 to 4	26.0	24.8–27.3	38.9	35.6–42.4
5 or more	13.4	12.5–14.4	50.1	46.4–53.8

Total			30.8	28.8–33.0

^a^ Percentage adjusted by sample weighting and post-stratification by age and gender.

^b^ Non-adherence = low adherence according to the Brief Medication Questionnaire BMQ (2 or more positive answers).

Self-perceived health was strongly associated with low adherence to treatment, i.e., the likelihood of low adherence was about three times higher in those with poor or very poor self-perceived health. Regarding the number of chronic diseases, among those with three or more, prevalence of low adherence was about double that of individuals with only one disease. Those who reported limitations caused by chronic diseases had about 80.0% lower adherence to treatment.

Regarding the therapy regimen used to treat reported chronic diseases, those who were taking five or more drugs had 2.4 times lower adherence to treatment than those who used only one drug.

Table 4 features the results of the crude and adjusted analyses. In the crude analysis, the variables skin color, marital status, economic status and seeing a doctor to treat chronic diseases were not statistically significant and therefore did not enter the adjusted analysis model. After adjustment for potential confounders in the multivariate analysis, the variables gender, level of education, number of hospitalizations in the last 12 months and self-perceived health lost their statistical significance. The following remained associated with low adherence to treatment for chronic diseases after the adjusted analysis: age, region, health insurance, number of doctors seen to treat chronic diseases, free access to therapy, number of emergency visits in the last 12 months, number of chronic diseases, limitation caused by disease and use of medicines.

**Table 4:** Crude and adjusteda,b prevalence ratios of low adherence to treatment for chronic diseases by adults aged 20 or over in Brazil, by analyzed variables. PNAUM, Brazil, 2014. (N = 11,842)

Variable^c^	Crude analysis			Adjusted analysis		
	
	PR	95%IC	p^d^	PR	95% IC	P^d^
Level 1						
Demographic characteristics						
Gender			0.017			
Male	Ref					
Female	1.13	1.02–1.25				
Age (years)			< 0.001			< 0.001
20-39	1.34	1.16–1.56		1.64	1.39-1.93	
40-59	1.05	0.95–1.16		1.16	1.04-1.29	
≥ 60	Ref					
Skin color			0.383			
White	Ref					
Non-white	1.04	0.94–1.16				
Marital status			0.409			
With partner	Ref					
Without partner	1.03	0.95–1.12				
Socioeconomic characteristics						
Level of education (years of study)			0.003			
No education	1.11	0.97–1.27				
1 to 8 years	0.89	0.81–0.99				
≥ 8 years	Ref					
Economic status ABEP^d^			0.372			
A/B	Ref					
C	0.98	0.86–1.12				
D/E	1.06	0.90–1.25				
Brazilian region			< 0.001			< 0.001
North	Ref					
Northeast	1.68	1.37–2.05		1.33	1.10-1.61	
Southeast	1.28	1.03–1.61		1.11	0.90-1.38	
South	1.18	0.96–1.44		0.98	0.81-1.18	
Midwest	1.56	1.26–1.94		1.21	1.00-1.47	
Health plan			0.102			0.031
Yes	0.91	0.82–1.01		0.89	0.81-0.99	
No	Ref					

Level 2						

Health system characteristics						
Visits doctor to treat chronic diseases			0,668			
Yes	Ref					
No	0.96	0.82–1.12				
Number of doctors visited to treat chronic diseases			< 0.001			< 0.001
One	Ref					
More than one	1.47	1.34–1.61		1.16	1.06-1.26	
Free access to drug therapy for chronic diseases			< 0.001			< 0.001
All free	Ref					
Any free	1.83	1.64–2.04		1.32	1.18-1.49	
None free	1.14	1.01–1.28		1.14	1.02-1.29	
Number of hospitalizations in the previous 12 months			< 0.001			
None	Ref					
1	1.15	0.99–1.34				
2 or more	1.73	1.44–2.08				
Number of emergency visits in the previous 12 months			< 0.001			< 0.001
None	Ref					
1	1.47	1.32–1.63		1.14	1.03-1.27	
2 or more	1.84	1.63–2.09		1.32	1.18-1.48	
Self-perceived health and morbidities						
Self-perceived health			< 0.001			
Very good	Ref					
Good	1.45	0.99–2.12				
Average	2.08	1.40–3.08				
Poor	2.69	1.78–4.07				
Very poor	2.88	1.91–4.32				
Number of chronic diseases (comorbidities)			< 0.001			< 0.001
1	Ref					
2	1.59	1.41–1.79		1.28	1.10-1.48	
3 or more	2.11	1.88–2.31		1.39	1.16-1.66	
Limitations due to chronic disease			< 0.001			< 0.001
No limitations	Ref					
Limitations	1.80	1.64–1.97		1.34	1.21-1.49	

Level 3						

Use of drugs						
Number of drugs used to treat chronic diseases			< 0,001			< 0,001
1	Ref					
2	1,28	1,12–1,46		1,08	0,93–1,26	
3 to 4	1,89	1,67–2,12		1,43	1,21–1,69	
5 or more	2,43	2,14–2,76		1,61	1,34–1,94	

^a^ Non-adherence = low adherence according to BMQ (2 or more positive answers).

^b^ According to the Brief Medication Questionnaire (BMQ).

^c^ Variables grouped by entry in the adjusted analysis model.

^d^ Wald test.

^e^ According to *Critério de Classificação Econômica Brasil* 2013 – ABEP. Available from: http//www.abep.org.

## DISCUSSION

Adherence is a multidimensional phenomenon determined by the interaction of a set of factors that affect people’s behavior and ability to follow treatment^22^. This study evaluated for the first time the factors associated with low adherence to treatment for chronic diseases in a representative sample of the Brazilian population aged 20 or more, contributing to build evidence on the subject to guide intervention strategies to improve treatment adherence among these patients.

About a third of the adult population showed low adherence to drug treatment for chronic diseases, a result similar to the systematic review that summarized data from international studies on the subject published over 50 years (1948 to 1998)^5^. Previous national studies showed great variability in prevalence, ranging from 17.0% to 63.5%^6^ ^,^ ^7^ ^,^ ^17^ ^,^ ^18^ ^,^ ^20^, but comparing results requires care because of the significant differences between studies in range of samples (local or regional), population subgroups, or focus on specific chronic diseases, such as high blood pressure.

The relationship between socioeconomic factors, such as income and education, and treatment adherence is widely investigated and previous studies have found an association between these variables and adherence, especially in chronic diseases^5^. In this study, low adherence to treatment was higher in individuals with lower levels of educationa, showing that this is a factor that must be considered in health care. Such patients require guidance regarding treatment to better understand the prescribed therapy regimens. On the other hand, economic status was not associated with the treatment of chronic diseases in Brazil.

Regarding demographic factors, the literature suggests that individuals who are young, male, and black show lower adherence to treatment ^7^ ^,^ ^9^. Our findings indicate that, among the Brazilian population, there is no significant difference between men and women, but younger people show lower adherence to treatment.

Residents of the Northeast and Midwest Regions had greater prevalence of low adherence to treatment than other regions, a result previously found by a study that evaluated the prevalence and factors associated with the non-use of continuous drugs among individuals who reported diagnosis of high blood pressure in the *Pesquisa Nacional de Domicílios* PNAD-2008^6^ (National Household Sample Survey).

Regarding the characteristics related to the health of individuals, very poor self-perceived health was positively associated with low adherence to treatment in patients treating chronic diseases. A meta-analysis described that patients with better self-perceived health have better adherence to treatment, which can help reduce the worsening of patients, especially those afflicted with chronic diseases^4^.

The demographic transition we are currently experiencing, with an increase in the number of chronic diseases, has led to a growing use of medicines, especially among older adults^20^. In this study, we found a strong association between higher number of chronic diseases and low adherence. The explanation is that the simultaneous treatment of many chronic health conditions can result in polypharmacy, complex regimens in which medicine is taken many times a day, involving drug risks and predisposition to non-adherence^10^ ^,^ ^22^. Wang et al. (2005) described a significant reduction in the use of antihypertensive drugs in older patients with high blood pressure who have a high prevalence of comorbidities, reinforcing the impact of polypharmacy in adherence to treatment for chronic conditions^21^.

Another factor described as one of the most important related to treatment adherence is medication costs^11^ ^,^ ^18^. A meta-analysis showed an 11.0% higher likelihood of non-adherence to medication in populations with health insurance who had to pay part of their medication costs, which may burden the public health system by increasing expenses from hospitalizations because of non-adherence to essential drugs^19^.

In Brazil, patients have free access through the Brazilian Unified Health System (SUS) to a list of essential medicines, with emphasis on the treatment of the most prevalent diseases, such as chronic diseases. However, a study that evaluated the availability of drugs in public health units in the country found low availability of drugs in all population strata^12^. In this study, the highest prevalence of low adherence to treatment was found among individuals who had to pay part of their treatment compared to those who had free access to all medicines needed to treat reported chronic diseases. This finding reinforces the fact that drugs not provided by SUS can lead users to abandon prescribed treatments for not being able to buy them in the private sector with their own resources^20^.

Regarding therapy regimens, the amount of prescribed drugs, the therapy schedule and the side effects are also associated with non-adherence^16^. The complexity of the therapy schedule, where the most relevant element is the number of prescribed drugs, also seems to contribute greatly to adherence to treatment^8^. In this study, individuals who used three or more drugs had a higher prevalence of low adherence to treatment, reinforcing this aspect as an important negative predictor of adherence to treatment.

Among the strategies to improve adherence are patient education, better treatment regimens and better communication between physicians and other health professionals and patients^15^. We noted in this study that individuals who reported seeing more than one doctor to treat their chronic diseases had a higher prevalence of low adherence to treatment, suggesting flaws in the whole care process. A recent systematic review shows that most of the current methods to improve adherence to treatment for chronic health problems are complex and ineffective. This shows the need for progress in this field, including improvement in the design of long-term viable interventions, objective adherence measures, and research capability, which should be sufficient to detect improvements in clinical outcomes of patients^14^.

The study’s limitations include the use of self-reporting to measure adherence to drug therapy, which is subject to measurement bias, and the actual cross-sectional design, which does not identify changes in health status, treatment regimens and other factors that can influence the behavior of patients’ adherence to treatment over time^4^. Moreover, the great variability of methods, tools and recall periods used to measure adherence limits the comparability of results. Despite the limitations, we were able to estimate in an unprecedented manner the factors associated with low adherence to drug therapy for chronic diseases in Brazil, contributing to the production of evidence to support the guidance of interventions addressing the subject in the country.

The results indicate that low adherence to drug treatment for chronic diseases in Brazil is relevant, and that regional and demographic differences and those related to patients’ health care and therapy regimen require coordinated action between health professionals, researchers, managers and policy makers.
